# Abnormal functional network connectivity mediates the relationship between depressive symptoms and cognitive decline in late-onset depression

**DOI:** 10.1017/S0033291725100706

**Published:** 2025-10-08

**Authors:** Zhidai Xiao, Ben Chen, Mingfeng Yang, Qiang Wang, Danyan Xu, Gaohong Lin, Pengbo Gao, Shuang Liang, Qin Liu, Jiafu Li, Xiaomin Zheng, Xiaomei Zhong, Yuping Ning

**Affiliations:** 1 Geriatric Neuroscience Center, The Affiliated Brain Hospital, Guangzhou Medical University, Guangzhou, China; 2 The First School of Clinical Medicine, Southern Medical University, Guangzhou, Guangdong Province, China; 3 Key Laboratory of Neurogenetics and Channelopathies of Guangdong Province and the Ministry of Education of China, Guangzhou Medical University, Guangzhou, China

**Keywords:** late-onset depression, cognitive decline, static functional network connectivity, dynamic functional network connectivity, functional magnetic resonance imaging

## Abstract

**Background:**

Late-onset depression (LOD) is featured by disrupted cognitive performance, which is refractory to conventional treatments and increases the risk of dementia. Aberrant functional connectivity among various brain regions has been reported in LOD, but their abnormal patterns of functional network connectivity remain unclear in LOD.

**Methods:**

A total of 82 LOD and 101 healthy older adults (HOA) accepted functional magnetic resonance imaging scanning and a battery of neuropsychological tests. Static functional network connectivity (sFNC) and dynamic functional network connectivity (dFNC) were analyzed using independent component analysis, with dFNC assessed via a sliding window approach. Both sFNC and dFNC contributions were classified using a support vector machine.

**Results:**

LOD exhibited decreased sFNC among the default mode network (DMN), salience network (SN), sensorimotor network (SMN), and language network (LAN), along with reduced dFNC of DMN-SN and SN-SMN. The sFNC of SMN-LAN and dFNC of DMN-SN contributed the most in differentiating LOD and HOA by support vector machine. Additionally, abnormal sFNC of DMN-SN and DMN-SMN both correlated with working memory, with DMN-SMN mediating the relationship between depression and working memory. The dFNC of SN-SMN was associated with depressive severity and multiple domains of cognition, and mediated the impact of depression on memory and semantic function.

**Conclusions:**

This study displayed the abnormal connectivity among DMN, SN, and SMN that involved the relationship between depression and cognition in LOD, which might reveal mutual biomarkers between depression and cognitive decline in LOD.

## Introduction

Late-onset depression (LOD) is one of the most prevalent psychiatric diseases in the older generation (Kok & Reynolds, 2017), typically manifesting with the initial depressive episode at the age of 55 or above (Dols et al., 2017; Geerlings & Gerritsen, 2017). LOD is characterized by impaired cognitive performance, in terms of memory, processing speed, visuospatial, executive, and semantic functions (Liu et al., 2018; Wang et al., 2021; Yin et al., 2015). These cognitive deficits still remain even though depressive symptoms remit (Cheng et al., 2020; Yin, Wang, Zhang, & Yuan, 2016). Besides, traditional medication and psychotherapies hardly improve depression-related cognitive deficits (Schneider et al., 2023). It should be noticed that LOD is considered as one of crucial factors of mild cognitive impairment (MCI) and Alzheimer’s disease (AD) (Diniz et al., 2015; Lauriola et al., 2018; Liu et al., 2024; Liu, Jiang, & Yuan, 2018), and they may experience worse performance and more rapid decline compared to those with early-onset depression (Hashem, M, Gomaa, & Khalaf, 2017; Ly et al., 2021). Therefore, it is necessary to unravel the underlying mechanisms of LOD and conduct tailored treatment of LOD with impaired cognition.

By functional magnetic resonance imaging (fMRI), several abnormalities of functional connectivity have been discovered in LOD, such as in hippocampal subregions (Wang et al., 2015), olfactory regions (Liu et al., 2022), and amygdala (Yue et al., 2013). However, various brain regions with different functions tightly communicate with each other for integrating information and realizing different functions as a large and complex scale of networks (Qiu et al., 2023; Van Den Heuvel & Hulshoff Pol, 2010). Examining functional network connectivity (FNC) is beneficial to discover altered organization among several brain regions, investigate pathophysiological characteristics in clinical studies, and identify mental diseases (Takamura & Hanakawa, 2017). The default mode network (DMN) and salience network (SN) have been found to be altered in various neuropsychological diseases. DMN, mainly consisting of posterior cingulate cortex (PCC), precuneus, and prefrontal cortex, is believed to involve self-referential processing, emotion regulation, and cognition (Hwang et al., 2016). SN, containing dorsal anterior cingulate cortex, insula, and so on, exerts a remarkable influence on interoceptive and emotional information (Bertocci et al., 2023). Both of them have been reported as significant linkages between cognition and emotion (Van den Stock et al., 2017), and their abnormalities with other networks have been discovered in patients with LOD. For instance, aberrant static FNC (sFNC) between DMN and SN was discovered to be associated with working memory and depression (Bertocci et al., 2023). sFNC of DMN in LOD individuals presented weaker connectivity between the posterior cingulate cortex and the right medial temporal lobe in the resting state but stronger in the task-based state, correlating with delayed recall (Wu et al., 2013). Decreased sFNC between DMN and cingulo-opercular network and its correlations with working memory and executive speed were also presented in LOD (Yin et al., 2016). However, sFNC of other cerebral networks and the correlation with depressive symptoms and more cognitive performances in LOD remain unclear.

Dynamic connectivity analysis is a novel method that reveals the correlations among various voxels as they fluctuate over time, particularly advantageous for capturing the dynamic interactions within the brain that are critical to understanding neural function and dysfunction (Rashid, Damaraju, Pearlson, & Calhoun, 2014). Dynamic functional network connectivity (dFNC), one of the dynamic analyses, may contribute to capturing transient signs of damage, focusing on network interactions, which is also favorable to distinguish patients from controls (Huang et al., 2024; Liu, Jiang, & Yuan, 2018; Xu et al., 2022). Through dFNC analysis, specific patterns of brain network connectivity observed over time, referred to as states, can be identified. This approach enables the detection of aberrant temporal characteristics, such as the number of transitions, representing shifts between distinct dynamic functional connectivity states, which signify the reliability of each state (Fiorenzato et al., 2019), which are not accessible for investigation in sFNC. Abnormalities of dFNC have been discovered in patients with schizophrenia (Long, Bhinge, Calhoun, & Adali, 2021), AD (Fu et al., 2019), and major depressive disorder (MDD) (Xu et al., 2022). In patients with acute mild traumatic brain injury, aberrance between DMN and sensorimotor network (SMN) could be witnessed in dFNC instead of sFNC (Lu et al., 2022). In a rich-club analysis of dynamic networks, global efficacy and path length of LOD displayed abnormality and correlated with processing speed (Mai et al., 2024). Additionally, dFNC of several brain networks, including DMN and SMN, exhibited abnormality in MDD patients compared with healthy individuals (Zhi et al., 2018). However, few studies of dynamic network characteristics and their association with various cognition domains in LOD were implemented.

In the current study, we aimed to explore the patterns of sFNC and dFNC in LOD individuals and their associations with different domains of cognitive performance. To translate the results to clinical practice, especially the diagnosis of LOD and the classification of LOD and healthy older adults (HOA) based on FNC (Shao et al., 2022; Zhang et al., 2023), we also applied support vector machine (SVM), a supervised classification algorithm for machine learning (Long & Yang, 2020). We hypothesized: (1) compared with HOA, LOD individuals show aberrant sFNC and dFNC across multiple networks, especially DMN and SN; (2) These abnormal network connections mediate the relationship between depressive symptoms and cognitive impairments in LOD. Understanding these abnormalities may help unveil crucial insights into the neural underpinnings of cognitive decline in LOD.

## Methods

### Participants

A total of 183 individuals were included in the current study, including 82 LOD and 101 HOA. All subjects, aged 55 years or above, were right-handed, of Han nationality, and were recruited from urban communities and the Affiliated Brain Hospital of Guangzhou Medical University, Guangzhou, China. All participants and their legal guardians provided written informed consent. The study protocol and assessments were approved by the Ethics Committees of the Affiliated Brain Hospital of Guangzhou Medical University.

All the LOD patients, experiencing the first depressive episode after the age of 55, were screened with the Diagnostic and Statistical Manual of Mental Disorders (DSM-IV) by two experienced psychiatrists. HOA met the criteria below: (1) had no history of depression and other psychiatric diseases; (2) demonstrated normal cognitive function. Based on medical history and physical examination, the criteria below were exclusionary for all participants included: (1) people who were unable to finish neuropsychological tests; (2) a history of other psychiatric diseases, such as schizophrenia and alcohol abuse; (3) serious neurological diseases, including brain tumors and stroke; (4) severe physical ailments, including severe cardiovascular diseases; (5) contraindications for MRI, such as metal implants.

### Clinical and neuropsychological assessments

Before accepting fMRI scanning, all subjects completed clinical assessments and various cognitive tests. The 17-item Hamilton Depression Scale (HAMD) and Geriatric Depression Scale (GDS) were used by psychiatrists to evaluate participants’ severity of depressive symptoms. Global cognition was also tested with the Mini-Mental State Examination (MMSE) and the activities of daily living scale (ADL). Besides, a battery of neuropsychological assessments included five different domains of cognitive functions: memory, with Working Memory Test (WMT) and Auditory Verbal Learning Tests (AVLT), Digital Span Tests (DST); information processing speed, with Symbol Digit Modalities Test (SDMT), Trail Making Test-Part A (TMT A) and Stroop Color and Word Test Part A (Stroop A); executive function, with Trail Making Test-Part B (TMT B) and Stroop Color and Word Test Part C (Stroop C); language, Boston Naming Test (BNT) and Verbal Fluency Test (VFT); visuospatial function, four-point scoring Clock Drawing Test (CDT4) and Rey–Osterrieth Complex Figure Test (Rey-O).

### MRI data acquisition and preprocessing

All participants accepted fMRI scanning in the Affiliated Brain Hospital of Guangzhou Medical University, using the same fMRI scanning machine (3.0 Tesla, Philips Achieva). The resting-state MRI data of whole brains were obtained with a single-shot gradient echo-planar imaging (EPI) sequence in 8 minutes. The parameters of scanning comprised: an echo time (TE) of 30 ms, a repetition time (TR) of 2,000 ms, a flip angle (FA) of 90°, acquisition of 33 slices with a thickness of 4 mm, a matrix size of 64 × 64, and a field of view (FOV) measuring 220 × 220 mm.

The fMRI data were preprocessed by using Data Processing and Analysis for Brain Imaging (DPABI) version 4.3 (https://rfmri.org/content/dpabi-v43-dpabisurf-v13-and-dparsf-v50-were-released) based on Statistical Parametric Mapping (SPM) (https://www.fil.ion.ucl.ac.uk/spm/software/spm12/) in Matlab (R2019b) software (Li, Lu, & Yan, 2020). First, the first 10 time points were eliminated. Second, slice-timing correction and realignment for head motion were performed. Subjects with maximum displacement over 2.0 mm or rotation over 2.0 degrees were excluded. The calculation of head motion parameters revealed no significant difference between the two groups (*p* = 0.522). Third, the realigned data were normalized by using EPI and resampled into 3 × 3 × 3 mm^3^. Finally, the data were smoothed with a 6 mm full-width at half maximum (FWHM) Gaussian kernel.

### Independent components and network identification

The preprocessed data were subjected to group independent component analysis (ICA) applying GIFT (version 3.0 b) toolbox (http://mialab.mrn.org/software/gift) in Matlab. First, principal component analysis (PCA) was utilized to reduce noise and dimensions, as well as simplify the complexity of computation. Second, we used the Infomax algorithm to estimate independent components (ICs), and eventually, 32 ICs were decomposed. To ensure the reliability and stability of the extracted ICs, ICASSO was utilized by repeating the ICA algorithm 100 times (Himberg, Hyvärinen, & Esposito, 2004). Additionally, spatial maps and time courses were back-reconstructed and then converted into z values with Fisher’s transformation.

The components were considered based on expectations that networks should display peak activations in grey matter, low spatial overlap with vascular, ventricular, motion, and susceptibility artifacts (Allen et al., 2014). We excluded 21 ICs and retained 11 ICs located in the cerebral cortex, given their critical role in cognition and emotion. According to anatomical and functional properties, 11 ICs were identified and classified into eight meaningful networks, such as the default mode network and the salience network, supported by previous studies (Dautricourt et al., 2022; Li et al., 2022; Thomas Yeo et al., 2011; Xu et al., 2022) by two investigators. For post-processing, all subject-specific time courses were detrended and despiked using 3dDespike in AFNI (Fiorenzato et al., 2019). We also applied a fifth-order Butterworth low-pass filter with a high-frequency cutoff of 0.15 HZ to filter and regress out translational and rotational head movement parameters (Fiorenzato et al., 2019).

### sFNC analysis

The static inter-network connectivity analysis was conducted based on the selected 11 ICs in the multivariate analysis of covariance (MANCOVA) toolbox v1.0 within GIFT, including age, sex, years of education, and mean framewise displacement (FD) as covariates. A matrix (183 subjects × 55 correlations) of sFNC Pearson correlations was obtained, and the values were Fisher’s z-transformed for further statistical analysis.

### dFNC analysis

The sliding window approach was implemented in the dFNC analysis, with a window size of 20 TRs (40s), which has been considered as the most popular, a step size of 1 TR (2s), and a 3 TRs Gaussian window alpha (Fu et al., 2019). We conducted L1-regularisation with 100 repetitions in the graphical least absolute shrinkage and selection operator (LASSO) algorithm. In addition, to determine the number of states, we applied K-means clustering analysis using squared Euclidean distance and finally estimated four clusters (*k* = 4) according to the elbow criteria. Temporal properties, including mean dwell time (DT), fraction time (FT), and number of transitions (NT), as well as matrices of dFNC Pearson correlations in different states, were acquired for further statistical analysis.

### Support vector machine

To further assess the classification from LOD and HOA and explore the strengths of features, we conducted support vector machine (SVM) via LIBSVM (https://www.csie.ntu.edu.tw/~cjlin/libsvm) in Matlab (Chang & Lin, 2011). The remarkable differences of sFNC and dFNC were revealed after statistical analyses were employed. A grid search was utilized to figure out the optimal penalty parameter (C) and radial basis function kernel parameter (γ) for enhancing the effect of the model (Chao & Horng, 2015; Ting et al., 2020), resulting in *C* = 10 and *γ* = 0.01. The values of area under the curve (AUC), sensitivity, accuracy, specificity, and weights of each feature contributed would be calculated, and the receiver operating-characteristic (ROC) curve was plotted, representing the diagnostic performance (Zheng et al., 2021).

### Statistical analyses

Demographic, clinical, and neuropsychological data were calculated in the Statistical Package for the Social Sciences (SPSS) version 26.0 software. After taking a normality test, we performed a two-sample *t*-test in normally distributed data and a Mann-Whitney test in abnormally distributed one in continuous data, and applied the Chi-square test in categorical data. The level of significance *p*-value was set at 0.05 (two-tailed).

Temporal properties in dFNC, including DT, FT, and NT, were calculated by analysis of covariance (ANCOVA) to assess group differences, with adjustment for age, sex, years of education, and mean FD. For the matrices of sFNC and dFNC, group differences were assessed using the Network-Based Statistic (NBS) v1.2 software (https://www.nitrc.org/projects/nbs), with 5000 permutations and a significance threshold of *p* < 0.05 (Feldmann et al., 2020). Age, sex, years of education, and mean FD were included as covariates.

In addition, using partial correlation analysis and false discovery rate (FDR) correction, the matrices of FNC and temporal properties of dFNC were calculated to assess the associations with depressive scales and various neuropsychological examinations, adjusted for age, sex, years of education, and mean FD. Furthermore, mediation models of depressive scales, cognitive tests, and FNC strength with significant difference were constructed by PROCESS in SPSS, with the four mentioned covariates, as well as a 95% confidence interval for the output and 5000 bootstrap samples (Chen et al., 2023).

## Results

### Demographic, clinical, and neuropsychological characteristics

The demographic, clinical, and neuropsychological information was displayed in Table 1. No obvious differences were discovered in age and head motion data. But the LOD group displayed a lower proportion of males (*p* = 0.015) and shorter educational attainment (*p* < 0.001) compared to the HOA group. All depressive and cognitive assessments showed remarkable discrepancies between the two groups (*p* < 0.001). To be specific, patients with LOD exhibited higher scores on depression scales and lower scores on MMSE, ADL, memory tests, SDMT, and language and visuospatial tests. They also spent much longer time on STROOP A, TMT A, STROOP C, and TMT B.

**Table 1.** Demographic, clinical, and neuropsychological data of all participants

Parameter	LOD (n = 82)	HC (n = 101)	*p* value
Demographic characteristics			
Sex (male%)	17(20.7%)	38(37.6%)	0.015^a^
Age (years)	66.0(7.0)	66.5(7.0)	0.947^c^
Education (years)	9.0(6.0)	11.0(4.3)	*<*0.001^c^
Clinical information of depression			
Age of onset (years)	60.0±9.0	–	–
Duration (years)	2.0±3.0	–	–
Numbers of episodes	1.0±1.0	–	–
Head movement parameter			
Mean FD (mm)	0.049(0.037)	0.052(0.042)	0.322^c^
Depressive symptoms			
HAMD	6.5(11.0)	1.0(3.0)	<0.001^c^
GDS	4.0(6.0)	1.0(2.0)	<0.001^c^
Daily life function			
ADL	14.0(0)	14.0(0)	<0.001^c^
Global cognition			
MMSE	24.5(5.0)	28.0(2.0)	<0.001^c^
Memory			
WMT	4.0(3.0)	7.0(3.0)	<0.001^c^
AVLT-I	16.4±5.49	21.0(7.0)	<0.001^c^
AVLT-S	5.0(4.0)	8.0(3.0)	<0.001^c^
AVLT-L	4.17±2.95	7.5(3.0)	<0.001^c^
AVLT-R	4.0(5.0)	7.0(3.0)	<0.001^c^
FDST	9.5(4.0)	11.0(3.0)	<0.001^c^
BDST	5.0(2.0)	7.0(3.0)	<0.001^c^
Information processing speed			
SDMT	26.5±10.4	36.8±6.3	<0.001^b^
STROOP A (s)	32.0(10.0)	27.0(7.0)	<0.001^c^
TMT A (s)	60.0(29.0)	44.0(22.0)	<0.001^c^
Executive function			
TMT B (s)	78.5(44.0)	56.0(27.0)	<0.001^c^
STROOP C (s)	90.5(48.0)	74.0(26.0)	<0.001^c^
Language			
BNT	20.0(4.0)	24.0(3.0)	<0.001^c^
VFT	13.5±6.0	16.0±5.0	<0.001^b^
Visuospatial ability			
Rey-O	25.0(7.5)	29.0(3.3)	<0.001^c^
CDT4	3.0(1.0)	4.0(0)	<0.001^c^

*Note:* “(s)” denotes “seconds,” indicating the time taken to complete these tests.

Abbreviations: HAMD, ‘Hamilton Depression Rating Scale’; GDS, ‘Geriatric Depression Scale’; MMSE, ‘Mini-Mental State Examination’; ADL, ‘Activities of Daily Living scale’; FDST, ‘Forward Digital Span Test’; BDST, ‘Backward Digital Span Test’; WMT, ‘Working Memory Test’; AVLT-I, ‘Immediate recall of Auditory Verbal Learning Test’; AVLT-S, ‘Short-term delayed recall of Auditory Verbal Learning Test’; AVLT-L, ‘Long-term delayed recall of Auditory Verbal Learning Test’; AVLT-R, ‘Recognition of Auditory Verbal Learning Test’; SDMT, ‘Symbol Digit Modalities Test’; TMT A, ‘Part A of Trail Making Test’; TMT B, ‘Part B of Trail Making Test’; STROOP A, ‘part A of Stroop Color and Word Test’; STROOP C, ‘part C of Stroop Color and Word Test’; BNT, ‘Boston Naming Test’; VFT, ‘Verbal Fluency Test’; Rey-O, ‘Rey–Osterrieth Complex Figure Test’; CDT 4, ‘four-point scoring Clock Drawing Test’.

a Chi-square test.

b Two-sample *t*-test, with data expressed as mean ± standard deviation.

c Mann-Whitney Test, with data expressed as median (interquartile range).

### IC and networks

**Figure 1.**
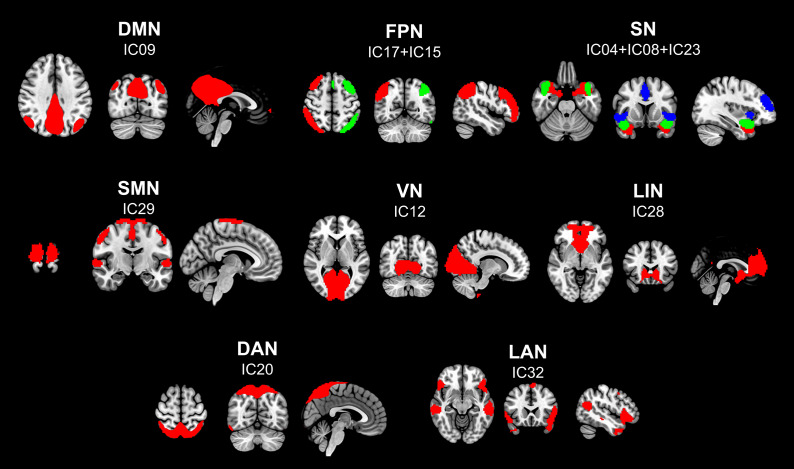
Spatial map of independent component. There are 11 independent components sorted into eight networks. Abbreviations: IC, ‘independent component’; DMN, ‘default mode network’; FPN, ‘frontoparietal network’; SN, ‘salience network’; SMN, ‘sensorimotor network’; VN, ‘visual network’; LIN, ‘limbic network’; DAN, ‘dorsal attention network’; LAN, ‘language network. Red color in FPN represents IC17, while green color represents IC15. In SN, red, green, and blue colors respectively represent IC04, IC08, and IC23.

11 ICs were divided into eight networks (Figure 1): default mode network (DMN), frontoparietal network, salience network (SN), sensorimotor network (SMN), visual network, limbic network, dorsal attention network, and language network (LAN). The related components and specific peak coordinates were also listed in Table S1 (see supplementary materials for details).

### Aberrance in sFNC

**Figure 2.**
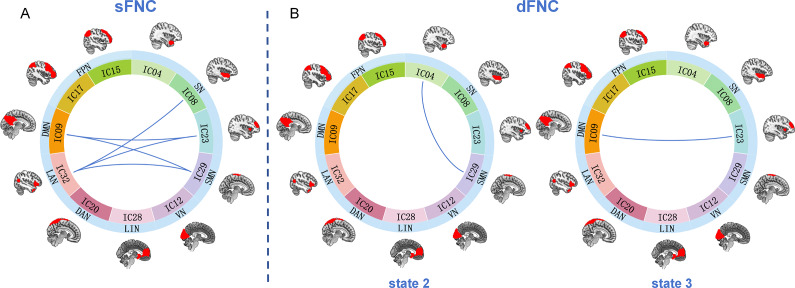
Visualization of declined FNC in LOD. (a) The chordal picture illustrates the reduced sFNC among several networks in LOD patients. (b) The dFNC of SMN-SN and DMN-SN, respectively reduced in states 2 and 3. The blue line represents decreased connectivity in LOD patients, with no increased connectivity discovered. Abbreviations: IC, ‘independent component’; DMN, ‘default mode network’; FPN, ‘frontoparietal network’; SN, ‘salience network’; SMN, ‘sensorimotor network’; VN, ‘visual network’; LIN, ‘limbic network’; DAN, ‘dorsal attention network’; LAN, ‘language network.

In terms of sFNC strength, remarkable decline was witnessed in DMN-SN (*p_NBS_ <* 0.001), DMN-SMN (*p_NBS_ <* 0.001), SN-LAN (IC08-IC32, *p_NBS_ <* 0.001), SN-LAN (IC23-IC32, *p_NBS_ <* 0.001), and SMN-LAN (*p_NBS_ <* 0.001) in LOD compared to HOA (Figure 2A).

### Aberrance in dFNC

**Figure 3.**
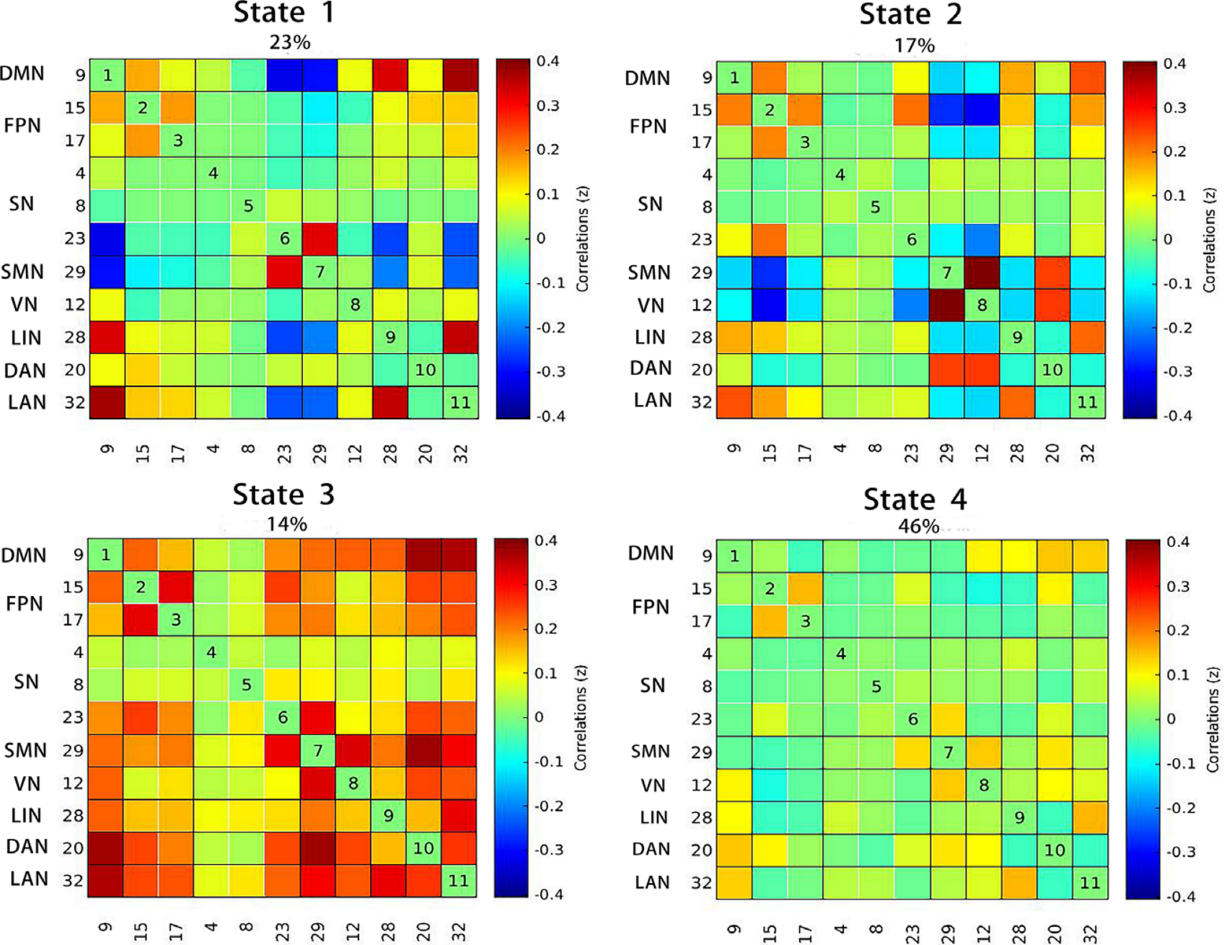
The correlation matrices and the percentage of occurrence with each state. There were four states identified by clustering analysis in dFNC. The percentage of all participants is shown above each state plot. The strength of connectivity among all networks is indicated by different colors. Abbreviations: DMN, ‘default mode network’; FPN, ‘frontoparietal network’; SN, ‘salience network’; SMN, ‘sensorimotor network’; VN, ‘visual network’; LIN, ‘limbic network’; DAN, ‘dorsal attention network’; LAN, ‘language network’.

For dFNC, four states were identified by clustering analysis (Figure 3). In state 1, LOD showed a trend toward longer FT compared with HOA (*p* = 0.027, *p_corrected_* = 0.055). While in state 3, LOD exhibited a tendency for shorter DT (*p* = 0.020, *p_corrected_* = 0.084) and FT (*p* = 0.019, *p_corrected_* = 0.078). No significant difference was found in NT (*p* = 0.159). Compared to the dFNC of HOA, weaker connectivity between SN (IC04) and SMN in LOD was discovered in state 2 (*p_NBS_* = 0.04). There was also a remarkably weaker dFNC between DMN and SN (IC23) in state 3 (*p_NBS_ <* 0.001) (Figure 2B).

### Classification results

**Figure 4.**
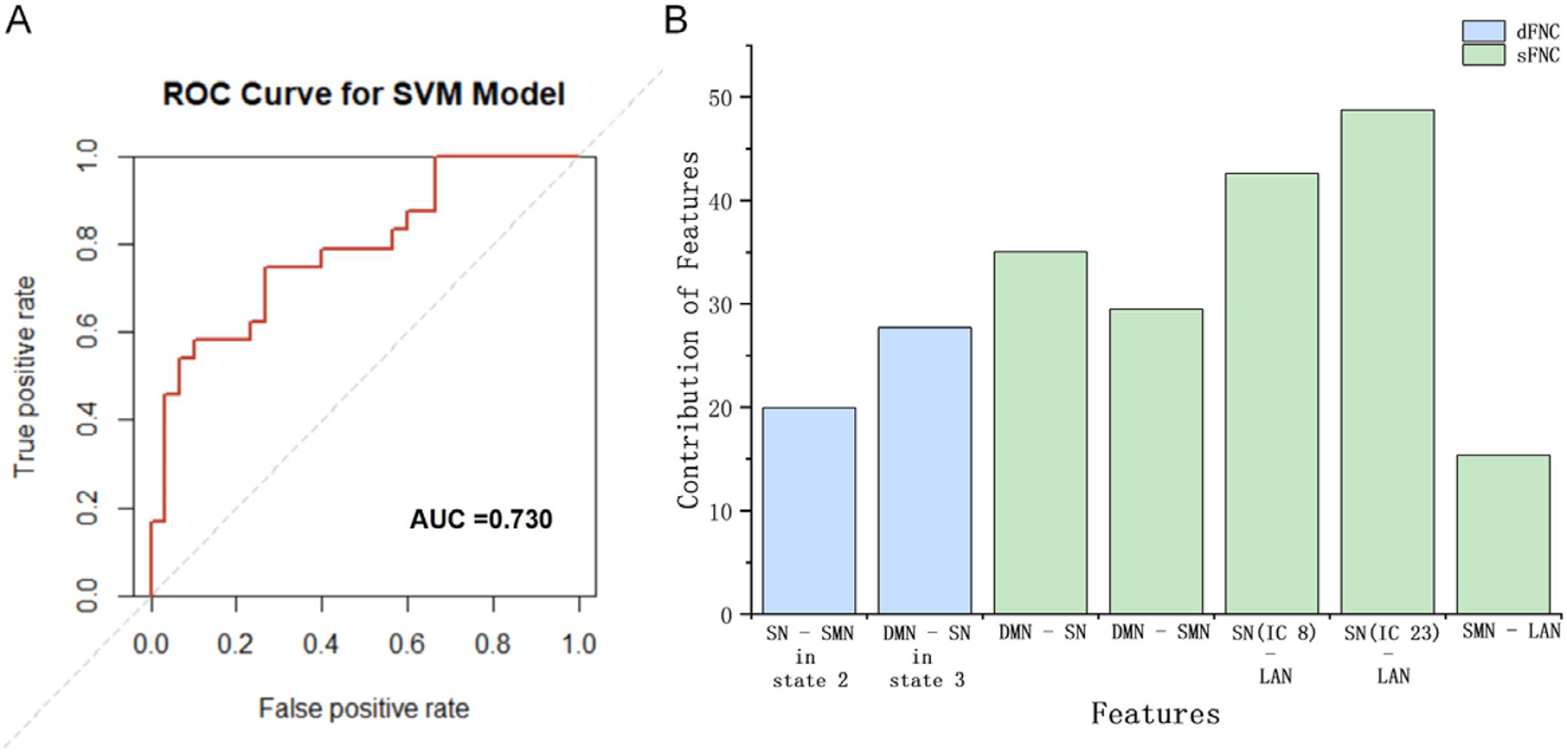
The performance of the SVM model within outcomes of sFNC and dFNC analyses. (a) The red line represents the ROC curve. (b) The bar chart illustrates the weights of dFNC of SN-SMN in state 2 and DMN-SN in state 3 (shown in blue bars) and sFNC of DMN-SN, DMN-SMN, SN (IC08)-LAN, SN (IC23)-LAN, and SMN-LAN (shown in green bars). Abbreviations: AUC, ‘area under the curve’; ROC, ‘receiver operating-characteristic’; SVM, ‘support vector machine’; dFNC, ‘dynamic functional network connectivity’; sFNC, ‘static functional network connectivity’; DMN, ‘default mode network’; SN, ‘salience network’; SMN, ‘sensorimotor network’; LAN, ‘language network’.

The AUC value of classification was at 73.0%, with a sensitivity of 63.4%, an accuracy of 67.0% and a specificity of 70.0% (Figure 4A). The connectivity between SN (IC 08 and IC 23) and LAN made the greatest contribution, followed by sFNC of DMN-SN, sFNC of DMN-SMN, dFNC of DMN-SN in state 3, dFNC of SN-SMN in state 2, and sFNC of SMN-LAN (Figure 4B).

### Correlation and mediation results

**Figure 5.**
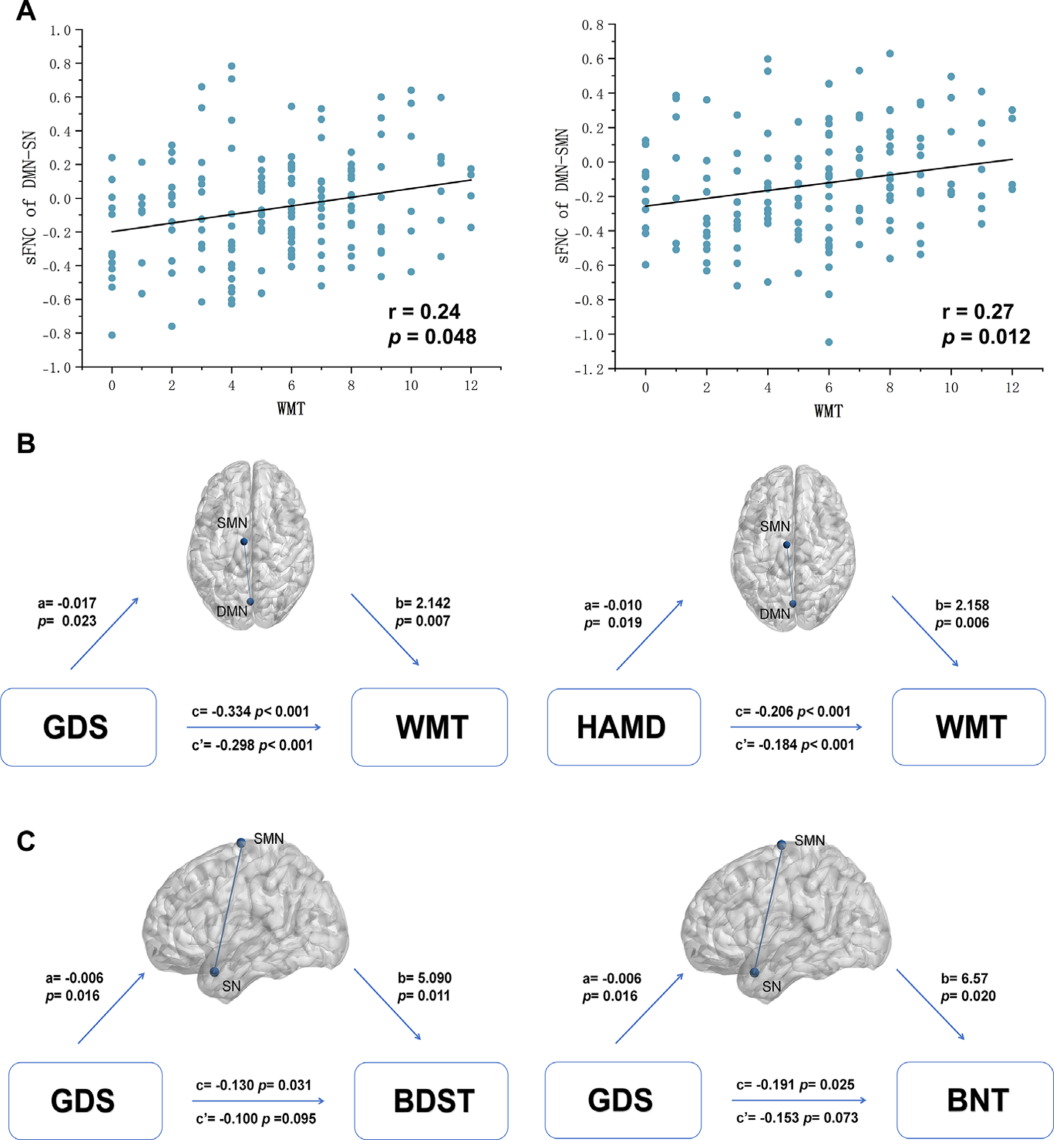
The correlations and mediation analyses of FNC. (a) sFNC of DMN-SN and DMN-SMN displayed positive associations with working memory. (b) The DMN-SMN presented partial mediation between the severity of depression and working memory. (c) The dFNC of SN-SMN in state 2 totally mediated the association between scores on the geriatric depression scale and the backward digital span test, as well as the Boston naming test. The blue dots respectively represent the regions of peak coordinates in DMN and SMN. “a” represents the influence on FNC by depression severity; “b” represents the influence on cognition by FNC; “c’ ” represents the direct effect; “c” represents the total effect. Abbreviations: WMT, ‘working memory test’; GDS, ‘geriatric depression scale’; HAMD, ‘Hamilton Depression Rating Scale’; BDST, ‘Backward Digital Span Test’; BNT, ‘Boston Naming Test’.

In sFNC, negative correlation could also be observed between SN-LAN and depressive severity. DMN-SN and DMN-SMN also exhibited significant correlations with memory-related scales in the overall sample (Supplementary Table S2). Notably, there was a significant positive correlation between scores of WMT and DMN-SN (r = 0.24, *p* = 0.048, FDR corrected), as well as DMN-SMN (r = 0.27, *p* = 0.012, FDR corrected) (Figure 5A). Moreover, the connectivity of DMN-SMN partially mediated the association between depressive symptoms and WMT (Figure 5B). Specifically, the total effect of GDS on WMT was −0.334 (*p <* 0.001, lower limit of confidence interval (LLCI) = −0.477, upper limit of confidence interval (ULCI) = −0.191) and the direct effect was −0.298 (*p <* 0.001, LLCI = −0.440, ULCI = −0.156). In the mediation between HAMD and WMT, the total effect was −0.206 (*p <* 0.001, LLCI = −0.286, ULCI = −0.126) and the direct effect was −0.184 (*p <* 0.001, LLCI = −0.264, ULCI = −0.105).

In the temporal properties of dFNC, FT in state 3 was negatively correlated with GDS score across all participants (Supplementary Table S3). The dFNC of DMN-SN also exhibited a negative association with depression levels. Besides, the connectivity strength in dFNC of SN-SMN was linked to depressive symptoms and cognitive impairment, including global cognition, memory, executive function, language, and visuospatial ability (Table S4, see supplementary materials for details). Furthermore, SN-SMN connectivity totally mediated the correlation between scores of GDS and the performance on both BDST and BNT (Figure 5C). In the mediation between GDS on BDST, the total effect was −0.130 (*p* = 0.031, LLCI = −0.248, ULCI = −0.012) and the direct effect was −0.100 (*p* = 0.095, LLCI = −0.218, ULCI = 0.018). The total effect of GDS on BNT was −0.191 (*p* = 0.025, LLCI = −0.357, ULCI = −0.025) and the direct effect was −0.153 (*p* = 0.073, LLCI = −0.320, ULCI = 0.015).

## Discussion

Our study applied MRI and neuropsychological tests to investigate the sFNC and dFNC of LOD patients and their correlations with cognition. To the best of our knowledge, it is the first research to explore LOD in the dual dimensions of FNC. The noticeable results mainly appeared in DMN, SN and SMN: (1) LOD displayed decreased connectivity of DMN-SN and DMN-SMN in sFNC, and declined dFNC of SN-SMN in state 2 and DMN-SN in state 3; (2) The strength of functional connectivity within the DMN-SN and DMN-SMN networks is positively linked to cognitive functions, with DMN-SMN exhibiting mediation effect in the association between depressive symptoms and working memory performance, while dFNC of SN-SMN in state 2 demonstrated correlations and mediation effects across various cognitive domains; (3) In SVM model, the connectivity of SN-LAN and DMN-SN held the highest weight in sFNC and dFNC, respectively.

Our study revealed distinct temporal characteristics in LOD patients, particularly in states 1 and 3. The longer FT in state 1 may indicate that LOD patients spend more time in this state, potentially reflecting a tendency toward prolonged engagement of several network connections. This finding aligns with a previous study on MDD, where longer FT was observed in a weakly connected state characterized by low or neutral connectivity among networks (Xu et al., 2022). Conversely, the shorter FT and DT in state 3, a strongly connected state, suggest that LOD patients transition more quickly out of this state, possibly indicating reduced stability or adaptability in network dynamics. In our study, FT in state 3 was linked to depression symptoms, and state 3 also exhibited abnormal connectivity between the DMN and SN, further supporting the role of disrupted network interactions in LOD. Similar findings have been reported in other neurological conditions; for example, Parkinson’s disease patients with MCI spent less time in a strongly connected state (Fiorenzato et al., 2019). These may highlight the unique value of dFNC in uncovering temporal abnormalities missed by sFNC, providing new insights into LOD’s altered network stability and flexibility.

In the present study, we discovered several abnormalities of FNC in individuals with LOD, especially among DMN, SN, and SMN. Abnormal intra-DMN connectivity has been linked to rumination (Hamilton, Farmer, Fogelman, & Gotlib, 2015), depressive severity (Yan et al., 2019), and suicidal ideation (Ordaz et al., 2018), while SN dysfunction is associated with depression severity (Manoliu et al., 2013), suicidality (Ho et al., 2021), and cognitive impairment (Schimmelpfennig, Topczewski, Zajkowski, & Jankowiak-Siuda, 2023). Besides, SMN, which is primarily involved in sensory and motor processing (Dieterich & Brandt, 2024; Liao et al., 2024), has also been shown to influence cognition and emotion in depression (Ray et al., 2021), anxiety (Kim & Yoon, 2018), and memory (Hsu et al., 2023; Iordan et al., 2021). Importantly, within-network dysconnectivity in these networks may contribute to their impaired inter-network communication. Abnormal intra-DMN connectivity, particularly hyperconnectivity between the hippocampus and medial prefrontal cortex, has been linked to rumination and may lead to excessive connectivity between the DMN and FPN (Kaiser, Andrews-Hanna, Wager, & Pizzagalli, 2015). Similarly, SN serves as a critical switch between the central executive network and DMN (Goulden et al., 2014; Manoliu et al., 2013), indicating that disruptions within the SN may impair its regulatory role in inter-network communication. Thus, intra-network dysconnectivity within the DMN, SN, and SMN may collectively disrupt their inter-network interactions, contributing to the cognitive and emotional deficits observed in LOD.

We initially identified a notable reduction in both sFNC and dFNC of DMN-SN in the LOD group. This finding, which significantly showed a great contribution in SVM, aligned with prior studies on connectivity patterns in depressive disorders across various age groups. Depressed adolescents with non-suicidal self-injury behaviors show lower sFNC of DMN-SN (Santamarina-Perez et al., 2019), while the reduced dFNC of DMN-SN is also witnessed in young adults with MDD (Xu et al., 2022). Similarly, in an electroencephalography research, the overall dynamic variability of DMN-SN connectivity strength in individuals with depressive disorder is lower than that in the normal (Lin et al., 2023). Besides, directed connectivity between DMN and SN can be inferred by the dynamic causal model (DCM). Participants with depression history exhibit greater directed connectivity from DMN to SN and less connectivity from SN to DMN (Guha, Yee, Heller, & Miller, 2021). Declined connection from SN to DMN is also found in depressed adolescents (Willinger et al., 2024). Interestingly, there is a decreased effective connectivity from posterior cingulate cortex (a part of DMN) to left insula (a part of SN) while an enhanced connectivity from posterior cingulate cortex to right insula in MDD subjects after electroconvulsive therapy (Ten Doesschate et al., 2023), suggesting that DMN-SN connectivity may serve as a potential biomarker for treatment response and that directional network connectivity warrants further exploration. While these findings suggest DMN-SN abnormalities are common across depressive disorders, systematic differences across age subgroups remain unclear. Despite a lack of direct comparisons among different age groups, a study has suggested that functional connectivity in DMN declines more rapidly with age in MDD patients (Tang et al., 2022). Future studies should investigate whether LOD exhibits unique DMN-SN connectivity profiles, providing insights into depression’s pathophysiology throughout life.

The sFNC of DMN-SN also displayed a positive association with working memory, a critical cognitive function that has been commonly documented to show a decline in LOD (Pu et al., 2012; Riddle et al., 2017). It has been demonstrated that DMN-SN plays a vital role in emotional regulation (Funkhouser et al., 2022), working memory (Bertocci et al., 2023; Zhang et al., 2020) and attention (Bremer et al., 2022). In healthy participants, when working memory task load elevates, connectivity of DMN-SN remarkably increases, similar to our finding (Liang, Zou, He, & Yang, 2016). The sFNC of DMN-SN has also been reported to have positive correlations with Trail Making Tests (Putcha et al., 2016). Although we only discovered the correlation between sFNC of DMN-SN and memory in LOD, previous literature has displayed that the connectivity between DMN and SN involves both depression and multiple domains of cognition. Patients with post-stroke depression present a positive correlation between the connectivity of anterior DMN-SN and aphasic depression rating scale scores (Balaev, Orlov, Petrushevsky, & Martynova, 2018). In adolescents with ahedonia, higher levels of mind wandering are associated with stronger functional connectivity between DMN and SN (Webb et al., 2021). By DCM, higher connectivity from DMN to SN is associated with lower overall network activation and poorer task performance in depressed individuals (Guha et al., 2021). In terms of dFNC, negative association with depression and positive association with cognitive performance have been witnessed in Cushing’s disease patients with depression (Feng et al., 2023). Reduced dFNC between DMN and SN was also discovered in patients with AD, revealing that aberrant DMN-SN probably involves cognitive deficits (Dautricourt et al., 2022). Therefore, the abnormality of DMN-SN could be construed as a common mechanistic pathway that underlies both depression and multiple cognitive decline.

We also discovered that LOD showed reduced sFNC of DMN-SMN, which is not frequently highlighted in the realms of depression or cognitive studies. In patients with subthreshold depression, the weakened sFNC between DMN and SMN has been found (Liao et al., 2024). Intriguingly, another study found that AD patients present reduced DMN-SMN in dFNC while sFNC between these networks is elusive (Fu et al., 2019). Moreover, our findings indicated that sFNC of DMN-SMN, positively correlating with memory, might serve as a mediator in the relationship between depression and memory. This finding is consistent with the established notion that depression exerts a detrimental impact on working memory, evidenced by performance on working memory tests (Songco et al., 2023) and fMRI with n-back tasks (Yang et al., 2020; Yüksel et al., 2018). Additionally, it has been reported that connectivity of DMN-SMN involves various cognition domains, including creative cognition (Patil, Madathil, & Huang, 2021), motor function (Zang et al., 2023), and attention (Wee et al., 2018). An analogous finding has shown that AD patients present reduced connectivity of DMN-SMN in some dynamic states, relevant to impaired semantic ability (Fu et al., 2019). Hence, our study suggested that sFNC of the DMN-SMN could not only serve as a key mechanism underlying LOD but also potentially intensify the detrimental effect of depression on the memory decline.

Besides, our study displayed a reduction in dFNC of SN-SMN, an observation that is rarely reported in the literature. Among patients with mild traumatic brain injury and cognitive impairment, the dFNC of SN-SMN displayed weaker compared to controls (Lu et al., 2022), while in patients with late-life depression, the aberrance displayed in sFNC (Almdahl et al., 2023). Furthermore, in this study, dFNC of SN-SMN correlated with cognition, including memory, executive functions, language, and visuospatial abilities. Most notably, it mediated the impact of depression on both memory and language capabilities, indicating its potential role in LOD pathology and its detrimental effects on cognition.

Although there was no correlation between sFNC of SMN-LAN and neuropsychological tests in the present study, SVM indicated it contributed the most in diagnosis. A study in male obstructive sleep apnea with MCI displayed a reduced dFNC of SMN-LAN without association in Montreal cognitive assessment and held the view that the aberration indicates low receptivity of external stimuli (Li et al., 2022), which has also been discovered in MDD patients (Valt et al., 2022), consistent with our findings. It is probable that resting-state brain networks may not reflect the connections as distinctly as task-based networks, which in turn leads to no correlation between the SMN-LAN connectivity and neuropsychological scales in the current study.

The abnormal network connections in LOD likely reflect vital mechanisms of neuroimaging and involve underlying and complex neurobiological mechanisms. A previous study has reported abnormal functional connectivity between DMN and cingulo-opercular network in LOD associated with cognitive impairment, including disrupted executive function and semantic memory, probably resulting from the demyelination of the white matter (Yin, He, et al., 2016). Additionally, altered connectivity within the DMN has been linked to several neural mechanisms. For instance, in postpartum depression, abnormal DMN connectivity is likely related to synaptic signaling, neuronal projection, and neurotransmitter alterations (Chen et al., 2024). In insomnia with high depressive symptoms, reward responsivity impairments were associated with DMN hyperconnectivity and dysfunction of dopaminergic signaling, as well as several genetic and inflammation factors. Similarly, functional connectivity changes in the DMN following electroconvulsive therapy have been associated with specific genetic factors in MDD (Li et al., 2023). However, neuropathological mechanisms of connections among DMN and other networks in LOD remain poorly understood. Future research should aim to elucidate these mechanisms to provide a more comprehensive understanding of the neurobiological basis of LOD.

Furthermore, disrupted connectivity among multiple neural networks in LOD may serve as potential targets or a prediction in some neuromodulation therapies (Cocchi et al., 2018; Hallett et al., 2017). In a research conducted on healthy individuals, reduced connectivity between the DMN and the rostral and dorsal anterior cingulate cortex has also been observed after intermittent theta burst stimulation (Singh et al., 2020). Besides, a clinical trial shows that sFNC of DMN-SN increases in MDD after repeated transcranial magnetic stimulation associated with declined scores of a depression scale (Godfrey, Muthukumaraswamy, Stinear, & Hoeh, 2022). Consequently, these findings indicate that inter-network connectivity could be applied as targets and predictors in neuromodulation therapies and further stimulation researches are indispensable to explore the mechanisms of inter-network connectivity and potential treatment for LOD.

There are several limitations of the current study. First, it is a cross-sectional study that investigated mechanisms among brain networks, depression, and cognition in LOD. It would be advantageous to conduct real-world longitudinal studies with MRI and clinical assessments at one-year intervals over a several-year follow-up period, which allows us to track changes in brain network connectivity and their relationship with depressive symptom progression and cognitive decline across different treatment trajectories in LOD. Second, the current study does not provide specific directional connectivity among the three networks. Future studies could use DCM to elucidate interactions among the DMN, SN, and SMN. Additionally, our exploration was limited to resting-state MRI, lacking real-time investigation. Task-based fMRI coupled with cognitive tests could be considered. Furthermore, future research should focus on elucidating the pragmatic significance of MRI imaging features in clinical practice. Specifically, integrating functional connectivity analysis with structural MRI, such as cortical thickness (Tang et al., 2024) and voxel-based morphometry (Guo et al., 2024), could help identify robust imaging signatures that correlate with clinical symptoms and cognitive impairments in LOD. This multimodal approach would enhance our understanding of LOD neuropathology and support the development of clinically applicable biomarkers for early diagnosis, personalized treatment, and therapeutic monitoring.

## Conclusion

In conclusion, our study mainly suggested that individuals with LOD exhibited decreased sFNC in DMN-SN and DMN-SMN, as well as diminished dFNC of DMN-SN and SN-SMN. sFNC of the DMN-SN appeared to mediate the link between depressive symptoms and working memory capacity. Additionally, dFNC of SN-SMN could potentially mediate the effects of depression on various cognitive domains. This study may provide insights into the mechanisms of LOD and suggest the development of targeted neuromodulation therapies aimed at alleviating specific cognitive impairments.

## Supporting information

Xiao et al. supplementary material Xiao et al. supplementary material

## Supplementary material

The supplementary material for this article can be found at http://doi.org/10.1017/S0033291725100706.
