# Supplementary material for: Abnormal functional network connectivity mediates the relationship between depressive symptoms and cognitive decline in late-onset depression
Source: Psychol Med. 2025 Oct 8;55:e227. doi: 10.1017/S0033291725100706 (PMC12551583; doi:10.1017/S0033291725100706)
Supplement: Xiao et al. supplementary material [file S0033291725100706sup001.zip › TableS1.docx]

**Supplementary materials**

**Table S1. Extracted components and networks information**

| **Network** | **Component number** | **Peak coordinate** | | | **Brain Region of**  **Peak coordinate** |
| --- | --- | --- | --- | --- | --- |
|  |  | **X** | **Y** | **Z** |  |
| DMN | 09 | 0.5 | -74.5 | 36.5 | PCUN_L |
| FPN | 17 | -50.5 | 15.5 | 32.5 | IFG_L |
|  | 15 | 42.5 | -63.5 | 53.5 | Angular_R |
| SN | 04 | -35.5 | 11.5 | -26.5 | TP_L |
|  | 08 | -39.5 | 11.5 | -20.5 | TP_L |
|  | 23 | 0.5 | 23.5 | 29.5 | ACC_R |
| SMN | 29 | -6.5 | -11.5 | 77.5 | SMA_L |
| VN | 12 | 11.5 | -62.5 | 3.5 | Lingual_R |
| LIN | 28 | -0.5 | 51.5 | -5.5 | MOFC_L |
| DAN | 20 | 3.5 | -65.5 | 62.5 | PCUN_R |
| LAN | 32 | -47.5 | 21.5 | -11.5 | IOFC_L |

Abbreviations: DMN, default mode network; FPN frontoparietal network; SN, salience network; SMN, sensorimotor network; VN, visual network; LIN, limbic network; DAN, dorsal attention network; LAN, language network; L, left; R, right; PCUN, precuneus, IFG, inferior frontal gyrus; TP, temporal pole; ACC, anterior cingulate cortex; SMA, supplementary motor area; MOFC, medial orbital frontal cortex; IOFC, inferior orbital frontal cortex.
