# Supplementary material for: Abnormal functional network connectivity mediates the relationship between depressive symptoms and cognitive decline in late-onset depression
Source: Psychol Med. 2025 Oct 8;55:e227. doi: 10.1017/S0033291725100706 (PMC12551583; doi:10.1017/S0033291725100706)
Supplement: Xiao et al. supplementary material [file S0033291725100706sup001.zip › TableS2.docx]

|  | **ALL participants** | | |  | **LOD** | | |  | **HOA** | | |
| --- | --- | --- | --- | --- | --- | --- | --- | --- | --- | --- | --- |
|  | **r** | ***p*** | ***q*** |  | **r** | ***p*** | ***q*** |  | **r** | ***p*** | ***q*** |
| **DMN-SN** | | | | | | | | | | | |
| WMT | 0.24 | **0.002** | **0.017** |  | 0.26 | **0.042** | 0.29 |  | 0.061 | 0.57 | 0.67 |
| AVLT-I | 0.19 | **0.012** | **0.043** |  | 0.071 | 0.55 | 1 |  | 0.16 | 0.11 | 0.77 |
| **DMN-SMN** | | | | | | | | | | | |
| GDS | -0.19 | **0.016** | **0.032** |  | -0.014 | 0.91 | 0.91 |  | -0.11 | 0.33 | 0.33 |
| BDST | 0.21 | **0.01** | **0.036** |  | -0.082 | 0.52 | 1 |  | 0.19 | 0.08 | 0.19 |
| WMT | 0.27 | **<0.001** | **0.004** |  | -0.045 | 0.73 | 1 |  | 0.22 | **0.039** | 0.28 |
| AVLT-R | 0.19 | **0.021** | **0.029** |  | -0.080 | 0.53 | 1 |  | 0.09 | 0.41 | 0.71 |
| **SN-LAN** | | | | | | | | | | | |
| GDS | -0.23 | **0.004** | **0.009** |  | -0.14 | 0.25 | 0.50 |  | -0.050 | 0.64 | 0.64 |
| HAMD | -0.20 | **0.007** | **0.007** |  | -0.088 | 0.45 | 0.45 |  | -0.069 | 0.50 | 1 |

**Table S2. Correlations between sFNC and assessments of all participants and two groups**

*q* values represent FDR-corrected *p*-values.
Abbreviations: HAMD, Hamilton Depression Rating Scale; GDS, Geriatric Depression Scale; BDST, Backward Digital Span Test; WMT, Working Memory Test; AVLT-I, Immediate recall of Auditory Verbal Learning Test; AVLT-R, Recognition of Auditory Verbal Learning Test.
