# Supplementary material for: Abnormal functional network connectivity mediates the relationship between depressive symptoms and cognitive decline in late-onset depression
Source: Psychol Med. 2025 Oct 8;55:e227. doi: 10.1017/S0033291725100706 (PMC12551583; doi:10.1017/S0033291725100706)
Supplement: Xiao et al. supplementary material [file S0033291725100706sup001.zip › TableS3.docx]

|  | **ALL participants** | | |  | **LOD** | | |  | **HOA** | | |
| --- | --- | --- | --- | --- | --- | --- | --- | --- | --- | --- | --- |
|  | **r** | ***p*** | ***q*** |  | **r** | ***p*** | ***q*** |  | **r** | ***p*** | ***q*** |
| **FT in S3** |  |  |  |  |  |  |  |  |  |  |  |
| GDS | -0.18 | **0.022** | **0.044** |  | -0.14 | 0.27 | 0.55 |  | -0.075 | 0.49 | 0.98 |

**Table S3. Correlation between FT in S3 and score of GDS in all participants and two groups**

*q* values represent FDR-corrected *p*-values.

Abbreviations: FT, Fraction Time; GDS, Geriatric Depression Scale.
