# Supplementary material for: Abnormal functional network connectivity mediates the relationship between depressive symptoms and cognitive decline in late-onset depression
Source: Psychol Med. 2025 Oct 8;55:e227. doi: 10.1017/S0033291725100706 (PMC12551583; doi:10.1017/S0033291725100706)
Supplement: Xiao et al. supplementary material [file S0033291725100706sup001.zip › TableS4.docx]

|  | **ALL participants** | | |  | **LOD** | | |  | **HOA** | | |
| --- | --- | --- | --- | --- | --- | --- | --- | --- | --- | --- | --- |
|  | **r** | ***p*** | ***q*** |  | **r** | ***p*** | ***q*** |  | **r** | ***p*** | ***q*** |
| **SN-SMN in state 2** | | | | | | | | | | | |
| MMSE | 0.21 | **0.004** | - |  | 0.20 | 0.085 | - |  | 0.011 | 0.92 | - |
| GDS | -0.20 | **0.013** | **0.026** |  | -0.07 | 0.55 | 1 |  | -0.16 | 0.13 | 0.25 |
| BDST | 0.23 | **0.004** | **0.009** |  | 0.26 | **0.039** | 0.27 |  | 0.11 | 0.32 | 0.55 |
| WMT | 0.21 | **0.008** | **0.015** |  | 0.25 | **0.047** | 0.17 |  | 0.05 | 0.64 | 0.64 |
| AVLT-I | 0.17 | **0.023** | **0.032** |  | -0.03 | 0.81 | 0.81 |  | 0.15 | 0.14 | 0.49 |
| AVLT-S | 0.23 | **0.002** | **0.016** |  | 0.13 | 0.28 | 0.49 |  | 0.12 | 0.23 | 0.54 |
| AVLT-L | 0.22 | **0.003** | **0.010** |  | 0.07 | 0.56 | 0.80 |  | 0.17 | 0.10 | 0.73 |
| AVLT-R | 0.17 | **0.03** | **0.036** |  | 0.06 | 0.65 | 0.76 |  | 0.08 | 0.48 | 0.56 |
| TMTB(s) | -0.21 | **0.007** | **0.014** |  | -0.10 | 0.45 | 0.90 |  | -0.21 | 0.05 | 0.05 |
| BNT | 0.20 | **0.011** | **0.022** |  | 0.24 | 0.06 | 0.12 |  | 0.02 | 0.87 | 0.87 |
| CDT4 | 0.20 | **0.011** | **0.022** |  | 0.21 | 0.11 | 0.22 |  | 0.04 | 0.69 | 0.69 |
| **DMN-SN in state 3** | | | | | | | | | | | |
| HAMD | -0.20 | **0.008** | **0.016** |  | -0.04 | 0.74 | 0.74 |  | -0.17 | 0.10 | 0.20 |
| GDS | -0.20 | **0.011** | **0.011** |  | -0.06 | 0.63 | 1 |  | -0.10 | 0.35 | 0.35 |

**Table S4. Correlations between dFNC and assessments of all participants and two groups**

*q* values represent FDR-corrected *p*-values.
Abbreviations: HAMD, Hamilton Depression Rating Scale; GDS, Geriatric Depression Scale; MMSE, Mini-Mental State Examination; BDST, Backward Digital Span Test; WMT, Working Memory Test; AVLT-I, Immediate recall of Auditory Verbal Learning Test; AVLT-S, Short-term delayed recall of Auditory Verbal Learning Test; AVLT-L, Long-term delayed recall of Auditory Verbal Learning Test; AVLT-R, Recognition of Auditory Verbal Learning Test; TMT B, Part B of Trail Making Test; BNT, Boston Naming Test; CDT 4, four-point scoring Clock Drawing Test.
